# A High-Density Genetic Map with Array-Based Markers Facilitates Structural and Quantitative Trait Locus Analyses of the Common Wheat Genome

**DOI:** 10.1093/dnares/dsu020

**Published:** 2014-06-27

**Authors:** Julio Cesar Masaru Iehisa, Ryoko Ohno, Tatsuro Kimura, Hiroyuki Enoki, Satoru Nishimura, Yuki Okamoto, Shuhei Nasuda, Shigeo Takumi

**Affiliations:** 1 Laboratory of Plant Genetics, Graduate School of Agricultural Science, Kobe University, Nada-ku, Kobe 657-8501, Japan; 2 Core Research Division, Organization of Advanced Science and Technology, Kobe University, Kobe, Japan; 3 Bio Research Laboratory, TOYOTA Motor Corporation, Toyota, Aichi 471-8572, Japan; 4 Graduate School of Agriculture, Kyoto University, Kyoto 606-8502, Japan

**Keywords:** array-based genotyping, chromosomal synteny, high-density genetic map, next-generation sequencing, QTL analysis

## Abstract

The large genome and allohexaploidy of common wheat have complicated construction of a high-density genetic map. Although improvements in the throughput of next-generation sequencing (NGS) technologies have made it possible to obtain a large amount of genotyping data for an entire mapping population by direct sequencing, including hexaploid wheat, a significant number of missing data points are often apparent due to the low coverage of sequencing. In the present study, a microarray-based polymorphism detection system was developed using NGS data obtained from complexity-reduced genomic DNA of two common wheat cultivars, Chinese Spring (CS) and Mironovskaya 808. After design and selection of polymorphic probes, 13,056 new markers were added to the linkage map of a recombinant inbred mapping population between CS and Mironovskaya 808. On average, 2.49 missing data points per marker were observed in the 201 recombinant inbred lines, with a maximum of 42. Around 40% of the new markers were derived from genic regions and 11% from repetitive regions. The low number of retroelements indicated that the new polymorphic markers were mainly derived from the less repetitive region of the wheat genome. Around 25% of the mapped sequences were useful for alignment with the physical map of barley. Quantitative trait locus (QTL) analyses of 14 agronomically important traits related to flowering, spikes, and seeds demonstrated that the new high-density map showed improved QTL detection, resolution, and accuracy over the original simple sequence repeat map.

## 1. Introduction

Common wheat (*Triticum aestivum* L., genome constitution AABBDD) is an allohexaploid species that originated from interspecific hybridization between tetraploid wheat (*Triticum turgidum* L., AABB) and the wild diploid relative, *Aegilops tauschii* Coss. (DD).^1,2^ In common wheat, its large genome (17 Gb) and allopolyploidy have complicated the construction of high-resolution genetic maps. Diversity arrays technology (DArT), a microarray hybridization-based technique, has been developed in common wheat for high-throughput genotyping without relying on sequence information.^3^ DArT generates whole-genome fingerprints by scoring the presence versus absence of DNA fragments in genomic representations generated by the digestion of genomic DNA with a combination of two restriction endonucleases.^4^ Using the wheat PstI(TaqI) v2.6 array, 1,348 DArT markers were mapped in a hexaploid Synthetic W7984 × Opata M85 doubled haploid population.^5^ The most recent common wheat PstI(TaqI) v3.0 array comprises about 7,000 markers that are polymorphic in a wide range of wheat cultivars.^6^

Recent advances in next-generation sequencing (NGS) technology have made it possible to screen a huge number of polymorphic sites even in species without reference genome information. Single-nucleotide polymorphisms (SNPs) were discovered by sequencing of a complexity-reduced fraction of the tetraploid wheat genome^7^ or cDNA of common wheat,^8–10^ and have been used to develop high-throughput SNP-typing platforms such as BeadExpress,^7^ KASPar,^8^ and Infinium.^10^ Using an Illumina 9K iSelect SNP assay, 7,504 polymorphic loci were positioned in a consensus map constructed based on integrating information on seven common wheat mapping populations.^10^

Improvements in the throughput of NGS technologies made it possible to conduct SNP typing of an entire mapping population by direct sequencing, combining the process of polymorphism discovery and genotyping in a single experiment. In tetraploid and hexaploid wheat, sequence-based genotyping has been performed by sequencing of complexity-reduced genomic DNA^11–13^ or a targeted genomic region.^14^ The former, called complexity reduction of polymorphic sequences (CRoPS), restriction site-associated DNA sequencing (RAD-seq), or genotyping-by-sequencing (GBS),^15^ was applied to the development of high-density genetic maps ranging from >10,000^11^ to >400,000 markers^12^ in the hexaploid wheat mapping population. In the latter, the targeted genomic region (for example, exome) can be captured and re-sequenced.^14^ Sequence-based genotyping is cost-effective and faster than array-based platforms, but often have a significant amount of missing data due to the low coverage of sequencing.^15^

To efficiently construct a high-resolution map in common wheat with few missing data points, we developed a microarray-based polymorphism detection system using NGS data obtained from complexity-reduced genomic DNA samples of two common wheat cultivars, Chinese Spring (CS) and Mironovskaya 808 (M808), and genotyped 210 recombinant inbred lines (RILs) between the two cultivars. Moreover, quantitative trait locus (QTL) analysis was performed for 14 morphological and agronomically important traits using the high-density map constructed.

## 2. Materials and methods

### 2.1. Plant material

CS and M808 were selected because of the difference in growth habit, geographical origin and abiotic stress responses, and the availability of RILs derived from them. M808 was bred in Mironovska Institute, Ukraine. It is reported to be one of the most freezing tolerant wheat cultivars tested.^16^ In contrast, CS is a spring-type wheat landrace from Sichuan Province in China. A mapping population of 210 RILs derived from a cross between two common wheat cultivars, CS and M808, developed by Kobayashi *et al.*,^16^ was used in this study. The plants were grown individually in pots arranged randomly in a field of Kobe University (34°43′N, 135°13′E) in two seasons (2009–10 [Kobe10, indicating location and growing season] and 2011–12 [Kobe12]), and in a glasshouse of Kyoto University (35°2′N, 135°47′E) in the 2011–12 season (Kyoto12). Total DNA from each RIL and the parents was extracted from leaves using standard procedures.^16^

### 2.2. Library preparation and NGS

DNA samples (180 ng) of CS and M808 were digested first with *Pst*I (New England BioLabs, Inc., Ipswich, MA, USA) at 37°C for 1 h and then with *Bst*NI (New England BioLabs) at 60°C for 1 h. We selected this combination of restriction enzymes based on *in silico* analysis in rice, our experience in sugarcane,^17,18^ and its use in barley.^4^ Digested DNA was ligated to *Pst*I adapters (5′-CACGATGGATCCAGTGCA-3′ and 5′-CTGGATCCATCGTGCA-3′) using T4 DNA ligase (Nippon Gene, Tokyo, Japan) and ATP (Wako Pure Chemical Industries, Osaka, Japan). The ligation reaction was run at 16°C for 16 h and the ligase was inactivated by holding at 60°C for 20 min.

Ligated samples were PCR-amplified for 30 cycles using 5′-GATGGATCCAGTGCAG-3′ with PrimeSTAR HS DNA Polymerase (Takara Bio, Shiga, Japan) under the following conditions: 10 s at 98°C, 15 s at 55°C, and 60 s at 72°C. PCR products were purified using a MinElute PCR Purification Kit (Qiagen, Hilden, Germany). The constructed libraries were sequenced as 2 × 100 nt paired-end reads on the Genome Analyzer IIx instrument (Illumina, San Diego, CA, USA). The sequences were deposited to the DNA Data Bank of Japan (DDBJ) Short Read Archive (DRA001257).

### 2.3. Processing of raw reads and design of probes

First, trimming of adaptor and primer sequences and low-quality 3′ ends (quality score ≤2) from raw reads was performed using an in-house developed software. Reads with the length of <70 nt were discarded including pairs. CS and M808 libraries were *de novo* assembled separately using Velvet version 1.1.05.^19^ Velveth was run with a k-mer size of 65, and velvetg with expected coverage: ‘auto’, coverage cut-off: ‘auto’, insert length of 220 bp, minimum contig length of 100 bp, and allowing scaffolding. From contigs and unused reads (in assembly), tiling probes were designed with a *T*_m_ of ∼76°C and the length of 50–70 bp.

To construct a primary array, the designed probes were first aligned to filtered reads of CS and M808 using Bowtie version 0.12.7,^20^ where no more than three mismatches were allowed and up to 40 alignments were reported (-v 3, -k 40). CS-derived probes that met all the following five conditions were selected: (i) the number of aligned CS reads with no mismatch was ≥5, (ii) no aligned CS reads with 1–3 mismatches, (iii) no aligned M808 reads with a perfect match, (iv) no aligned M808 reads with one or two mismatches, and (v) the number of aligned M808 reads with three mismatches was ≤2. The last condition was included because this array-based polymorphism detection system has been demonstrated to accurately detect differences of more than three nucleotides.^17,18^ A similar selection was performed with M808 probes. In total, 223,000 probes from both CS and M808 were spotted in triplicate on a NimbleGen 3 × 1.4 M array (Roche Diagnostics, Mannheim, Germany).

### 2.4. Hybridization and construction of secondary array

To select probes for the construction of a secondary array, CS, M808, and 14 randomly selected RILs were genotyped. After digestion of total DNA with *Pst*I and *Bst*NI, adapter ligation, PCR amplification and purification of PCR products as described in the library preparation section, they were labelled with Cy3 using the NimbleGen One-Color DNA Labeling Kit (Roche Diagnostics) according to the manufacturer's instructions. Hybridization was performed at 42°C for 72 h on a NimbleGen Hybridization System (Roche Diagnostics). Arrays were scanned using a NimbleGen MS200 Microarray Scanner (Roche Diagnostics) and genotype calling was performed based on the signal intensities.

For selection of probes, a *χ*^2^ test was used to compute segregation of each probe in the 14 RILs. Those deviating from Mendelian 1 : 1 segregation at a 1% significance level were discarded. The secondary array was constructed using one selected probe per contig or read, consisting of 21,346 CS-derived probes, 21,205 M808-derived probes, and 2,303 probes for normalization. These 44,854 probes were spotted in triplicate on a NimbleGen 12 × 135 K array (Roche Diagnostics). This array was used for genotyping of 210 RILs and the two parental lines.

### 2.5. Genetic map construction

The genotyping data for 422 simple sequence repeat (SSR) markers were previously generated.^16^ A total of 140 barc, 1 cfa, 34 cfd, 18 gdm, 109 gwm, 4 hbd, 15 hbe, 41 hbg, and 60 wmc loci were used for chromosomal assignment of wheat array-based markers (WABMs) developed in this study. *VRN-D1* was mapped using primer sets 5′-CTGGTTGTCTGCCTCATCA-3′ and 5′-CTCTCTCCCCCTGCTAGT-3′, and a second copy of *Ppd-B1*^21^ using 5′-TAACTGCTCGTCACAAGTGC-3′ and 5′-CCGGAACCTGAGGATCATC-3′. Loci were assembled into linkage groups using Antmap version 1.2^22^ with the ‘all combination’ method and different threshold distances (5.75, 12.0, 17.5, and 25.0 cM). Loci were ordered using MapDisto version 1.7.7,^23^ and the genetic distances were calculated with the Kosambi function.^24^

### 2.6. BLAST searches of the mapped contigs and reads

To search repeat elements (REs) in the mapped contigs and reads, they were used as queries against the Munich Information Center for Protein Sequences (MIPS) RE database version 9.0 for Poaceae (ftp://ftpmips.helmholtz-muenchen.de/plants/REdat/) using the blastn algorithm. A blastn search was also performed against *Ae. tauschii*,^25^ *Triticum urartu*,^26^ and barley high-confidence genes and genomic sequences.^27^ Contigs and reads mapping to the A-genome were searched by the blastn algorithm against the *T. urartu* draft genome sequence, while those mapping to the D-genome were searched against the *Ae. tauschii* draft genome sequence.^25,26,28^ All BLAST hits were filtered with an *E*-value cut-off of 10^−5^ and hit length of ≥50 bp. The genetic map and the results of the RE search were represented in a circular plot using the Circos version 0.63 software.^29^

### 2.7. In silico digestion of the draft genome sequences of T. urartu and Ae. tauschii

Using an in-house PERL script, genomic scaffolds of *T. urartu* and *Ae. tauschii* were fragmented at *Pst*I and *Bst*NI sites, and those having only *Pst*I sites at both ends but not *Bst*NI sites were extracted. A blastn search was performed against the MIPS RE database version 9.0 for Poaceae using as a query the extracted *T. urartu* and *Ae. tauschii* genomic fragments with lengths between 65 and 6,000 bp (*E*-value <10^−5^ and hit length ≥50 bp).

### 2.8. In silico mapping to the physical map of barley

For reads and contigs mapping to the A- and D-genomes, a blastn search was first conducted against draft genomic sequences (*T. urartu* for the A-genome and *Ae. tauschii* for the D-genome)^25,26^ as described above. The obtained scaffolds were mapped based on a blastn search of the genes contained in these scaffolds against the barley genome (*E*-value <10^−5^ and hit length ≥50 bp). The A- and D-genome scaffolds without genes as well as reads and contigs mapping to the B-genome were searched against the barley genome using the same blastn parameters.

### 2.9. Phenotypic measurements

A list of traits, growing season, and location is presented in Supplementary Table S1. Heading time (HT) and flowering time (FT) were recorded as days after sowing. Maturation time (MT) was evaluated as the number of days that had passed when the ear neck turned yellow. HT, FT, and MT were measured for the three earliest tillers of each plant, and mean values were calculated. Grain-filling period (GFP) was defined as the number of days from flowering to maturation.

Spike-related traits (such as spike length [SL], number of spikelets per spike [SpN], SL per number of spikelets [SLperSpN], and top five spikelets length [T5SpL]) and culm length (CL) were assessed for the three earliest tillers of each plant before anthesis. Seed-related traits such as seed length (SdL), seed width (SdW), seed height (SdH), and seed length-to-width ratio (SdLperW) were measured using seeds on the central spikelets of the first, second, and third spikes. For each spikelet, only the components of the first and second florets were used. The mean of the replicated measurements over three spikes was calculated for each trait and used as the trait value in subsequent analyses. SdL, SdW, SdH, and SdLperW were evaluated using at least 10 seeds per line. For tiller number (TN), the number of tillers bearing ears was counted at harvest.

### 2.10. QTL analysis

QTLs were analysed by composite interval mapping using R/qtl package version 1.21-2^30^ for the new map and for that constructed by Kobayashi *et al.*^16^ using SSR markers. This SSR map was constructed using 412 SSR markers. First, QTL genotype probability was calculated using the function calc.genoprob with a step size of 1 cM and Kosambi map function. QTL analysis was performed with cim function using three marker covariates and Kosambi map function. The log-likelihood (LOD) score threshold was determined by computing a 1,000 permutation test for all traits. The percentage of phenotypic variation (PV) explained by a QTL for a trait was estimated using the fitqtl function in R/qtl.

## 3. Results

### 3.1. Construction of a microarray

**Figure 1.**
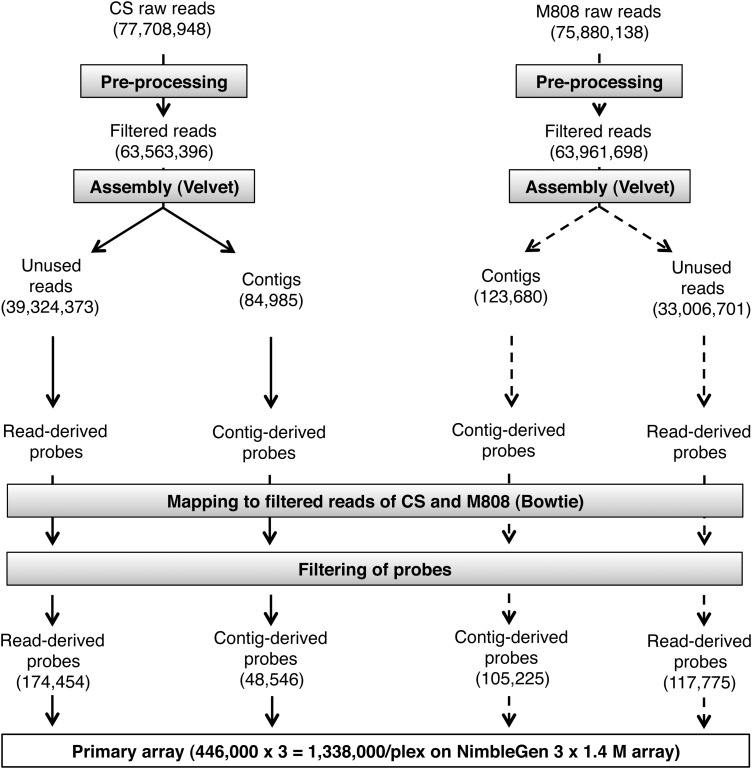
Schematic of primary array development. Raw reads obtained from sequencing complexity-reduced CS and M808 genomic DNA were pre-processed for quality filtering prior to assembly with Velvet. Tiling probes were designed from contigs and unused reads. To predict non-polymorphic probes, alignment to filtered CS and M808 reads was performed. The primary array was constructed by spotting 446,000 filtered probes in triplicate. See Materials and methods for more details.

To design probes for the development of a microarray, we sequenced complexity-reduced genomic DNA samples from two common wheat cultivars, CS and M808. Genome complexity was reduced using a combination of two restriction enzymes, *Pst*I and *Bst*NI. After ligation of adapters with sticky ends complementary to the 3′-overhang created by *Pst*I, only the DNA fragments having a *Pst*I site at both ends, with no *Bst*NI site, were amplified by PCR. After trimming and filtering of raw reads, 63,563,396 (6.04 Gb, genome coverage of 0.36× [Supplementary Table S2]) and 63,961,698 (6.09 Gb, 0.36×) reads were generated for CS and M808, respectively (Fig. 1). Both libraries were independently assembled using Velvet,^19^ yielding 84,985 contigs and 39,324,373 unused reads during assembly for CS and 123,680 contigs and 33,006,701 unused reads for M808.

From contigs and unused reads, tiling probes were designed. The probes were aligned with the filtered reads of CS and M808, and only the probes that predicted detection of ‘presence’ or ‘absence’ alleles were selected (see Section 2.3.). Thus, the primary array contained 1,338,000 probes per plex (446,000 probes in triplicate) on a 3 × 1.4 M array (Fig. 1) with a genome coverage of 4.51 × 10^−3^ fold.

The primary arrays were used for genotyping of CS, M808, and their 14 derived RILs, and of the 446,000 probes, 303,387 were polymorphic. Probes showing segregation distortion from a 1 : 1 ratio at a 1% significance level were discarded and only one probe per contig or read was selected. The final array contained 44,854 probes (including 2,303 probes for normalization, genome coverage of 7.45 × 10^−4^ fold) in triplicate on a 12 × 135 K array and was used for genotyping of CS, M808, and 210 RILs. We named these microarray probes as WABMs.

### 3.2. Construction of a high-density linkage map

In a previous study, a linkage map was constructed for 210 RILs of CS and M808 using 422 SSR loci.^16^ Of the 42,551 microarray probes, polymorphism was observed for 15,530 (36.5%) with a minor allele frequency of ≥10% among the 210 RILs. Missing data points were counted in each of the RILs and ranged from 17 to 1,074, with an average of 159.0 ± 153.0 (Supplementary Fig. S1A). To use high-quality data in further analyses, nine RILs with ≥465.1 (mean + 2 SD) missing data points were excluded. Missing data points were also counted for each marker and ranged from 0 to 42, with an average of 2.49 ± 5.84 (Supplementary Fig. S1B). Markers with >20 missing data points were excluded, and 14,464 markers were used for genetic map construction.

In total, 13,480 markers, including 13,056 WABMs, 422 SSRs, *VRN-D1*, and *Ppd-B1*, were grouped into 21 linkage groups. The remaining markers did not form linkage group or the linkage group was small (<10 cM) without chromosomal assignment. Of the mapped WABMs, 7,253 (55.6%) were probes derived from contigs. The probe sequences for these markers are presented in Supplementary Table S3. The number of total markers per chromosome ranged from 1,256 on chromosome 2B to 85 on chromosome 4D (Table 1). The number of markers with a non-redundant (nr) genotype pattern was 5,243, comprising 2,744 loci (markers with the same position on genetic map). The total map length was 5,093.12 cM, with an average marker interval of 0.38 cM when considering all markers, 0.97 cM per nr marker, and 1.86 cM per locus.

**Table 1.** Number of markers mapped to each chromosome of common wheat

Chromosome	Map length (cM)	Number of loci	Number of nr marker	Number of total marker
1A	239.87	168	241	571
1B	277.28	214	428	1,026
1D	137.82	76	131	353
2A	245.01	147	259	574
2B	309.67	201	428	1,256
2D	272.18	112	280	854
3A	228.25	128	233	548
3B	310.09	231	408	939
3D	256.95	70	161	401
4A	221.29	141	317	1,013
4B	158.60	77	145	506
4D	121.46	31	52	85
5A	319.07	129	206	473
5B	315.32	197	376	984
5D	292.74	73	142	272
6A	179.32	112	219	667
6B	241.01	167	363	940
6D	193.48	67	112	207
7A	288.54	178	338	771
7B	269.17	168	289	851
7D	216.00	57	115	189
Total	5,093.12	2,744	5,243	13,480
A genome	1,721.35	1,003	1,813	4,617
B genome	1,881.14	1,255	2,437	6,502
D genome	1,490.63	486	993	2,361

**Figure 2.**
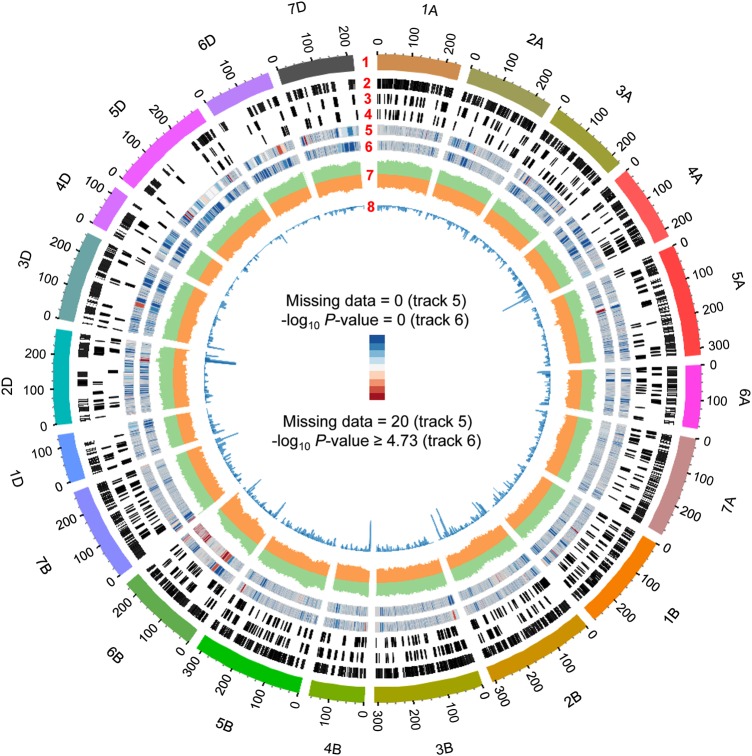
Distribution of WABMs, REs, missing data, and allele frequency on the constructed genetic map. From the outer to the inner circle, Track 1 shows the genetic map (scale in cM); Track 2 represents the distribution of sequence derived from genic regions; Tracks 3 and 4 represent, respectively, the distribution of retroelements and DNA transposons; Tracks 5 and 6, respectively, a heat map of missing data and *P*-values for segregation distortion; Track 7, the allele frequency of M808 (green) and CS (orange); and Track 8, a histogram of the number of markers (*y*-axis range 0–100).

Segregation distortion was computed for each marker and *P*-values of <1.82 × 10^−5^ (Bonferroni corrected *P*-value: 0.05/2,744 loci) from a *χ*^2^ test (1 : 1 ratio) were observed for markers of chromosomes 2B, 2D, 5A, 5B, 6B, and 7B (Fig. 2, Tracks 6 and 7; Supplementary Table S4). The greatest distortion was observed at the telomeric region of chromosome 6BL (235.8 cM), where the allele frequency was 89.5% for CS and 10.5% for M808.

### 3.3. Annotation of mapped contigs and reads

**Figure 3.**
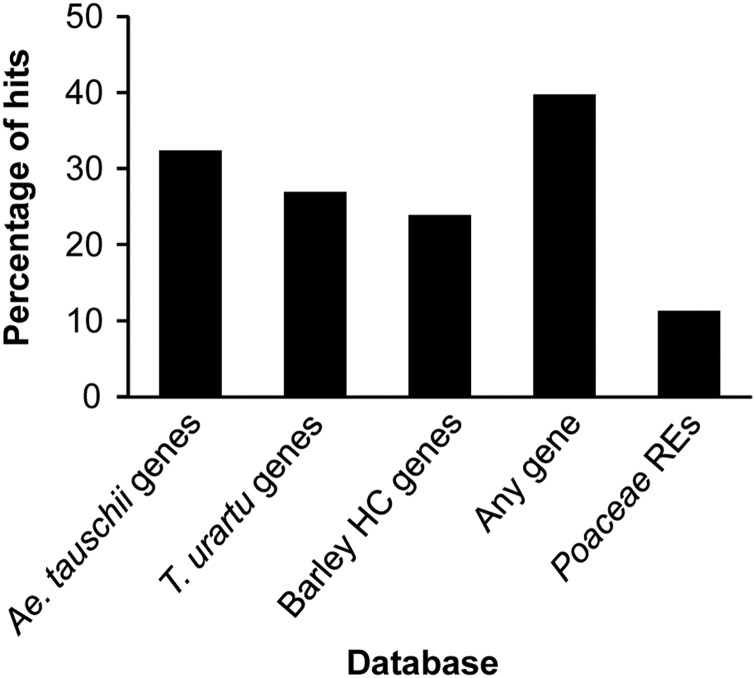
Histogram of annotated WABMs revealed by blastn analyses of genome databases. Reads/contigs anchored to the high-density genetic map were searched using the blastn algorithm against genes of *Ae. tauschii*, *T. urartu*, and barley and against Poaceae REs.

To annotate mapped contigs or reads, blastn searches were conducted against protein-encoding genes of *Ae. tauschii*, *T. urartu*, and barley, and against the RE database of the Poaceae of the MIPS. Of the 13,056 contigs/reads, 32.5, 27.0, and 23.9%, respectively, had hits to the *Ae. tauschii*, *T. urartu*, and barley genes (Fig. 3 and Supplementary Table S5). Overall, 39.8% of the contigs/reads had at least one hit in one of these three databases. These contigs/reads derived from genic regions were distributed all across the wheat genome and chromosomes (Fig. 2, Track 2; Supplementary Table S6).

REs were found in 11.4% of the mapped contigs/reads (Fig. 3). The proportion of DNA transposons and retroelements was similar (Table 2), and no bias in the distribution was found for these REs (Fig. 2, Tracks 3 and 4; Supplementary Table S6). The *CACTA* superfamily (present in 38.7% of the probes) was mainly found among the DNA transposons followed by the miniature inverted-repeat transposable element (MITE) family (25.6%) and *Mariner* superfamily (17.5%). Among the retroelements, *Gypsy* (30%), *Copia* (23%), and other (29%) long terminal repeat (LTR) retrotransposons were the predominant classes.

**Table 2.** REs found in the mapped contigs and reads

Repeat elements	Number of contigs (%)
DNA transposons	679 (45.8)
*CACTA*	263 (38.7)
MITE	174 (25.6)
*Tc1*/*Mariner*	119 (17.5)
Other DNA transposons	57 (8.39)
*Mutator*	37 (5.4)
*Harbinger*	29 (4.3)
Retroelements	659 (44.4)
LTR/*Gypsy*	196 (29.7)
Other LTRs	193 (29.3)
LTR/*Copia*	152 (23.1)
LINE	116 (17.6)
SINE	2 (0.3)
Others	146 (9.8)
Total	1,484

To test whether digestion with *Pst*I and *Bst*NI and selection of DNA fragments having only *Pst*I sites at both ends reduced the genomic fraction containing REs, *T. urartu* and *Ae. tauschii* genomic scaffolds were digested *in silico*. Overall, 136,189 and 106,724 fragments were, respectively, generated from the *T. urartu* and *Ae. tauschii* scaffolds. The size of A-genome fragments ranged from 6 to 23,779 bp with an average of 57.2 ± 551.7 bp, and that of D-genome fragments varied from 6 to 28,677 bp with an average of 44.9 ± 346.7 bp. The majority of these fragments were ≤80 bp (Supplementary Fig. S2). Because contigs and reads obtained from the NGS data of CS and M808 ranged from 65 bp to <6,000 bp, a blastn search of the *in silico* digested genomic sequences against the MIPS RE database was performed using as query the A- and D-genome-derived fragments between 65 and 6,000 bp. REs were found in 84,410 (76.1%) of the 110,984 A-genome fragments, and in 60,522 (69.2%) of the 87,466 D-genome fragments. In contrast to the sequences mapping to the linkage map, retroelements predominated on DNA transposons in the fragments derived from the *in silico* digestion of both *T. urartu* and *Ae. tauschii* genomes (Supplementary Table S7). REs found were mainly members of the *CACTA* superfamily of DNA transposons or *Gypsy* retroelements.

### 3.4. In silico mapping of the contigs/reads to the physical map of barley

**Figure 4.**
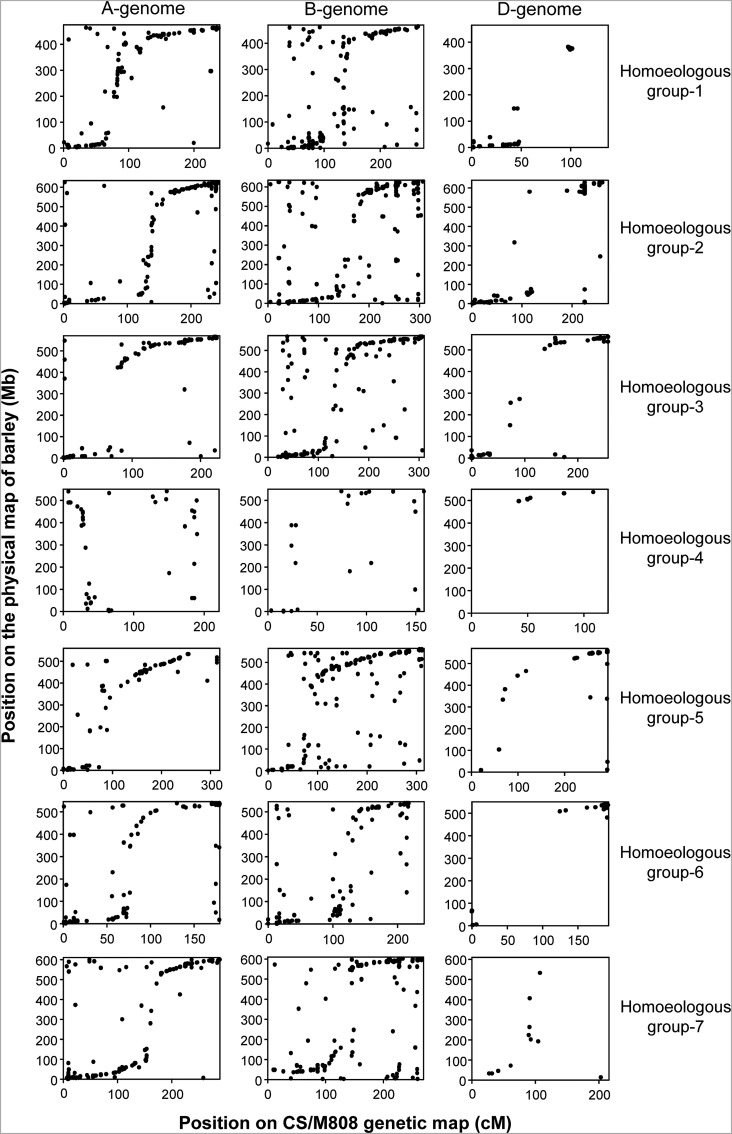
Relationship between the wheat genetic and barley physical maps. Based on blastn search sequences anchored to A- (left column), B- (centre), and D-genomes (right column) were aligned to the barley genome. Only the sequences mapped on the same homoeologous group are presented in each row. The *y*-axis indicates the position on the physical map of barley in Mb and the *x*-axis the position on the genetic map of wheat in cM.

To determine how many of the mapped sequences could be used for the analysis of synteny between wheat and barley (*Hordeum vulgare* L., H-genome) chromosomes, they were mapped *in silico* to the physical map of barley.^27^ For sequences mapping to the wheat A- and D-genomes, a blastn search was first conducted, respectively, against the *T. urartu* and *Ae. tauschii* draft genomic sequences;^25,26^ the scaffolds obtained were mapped based on a blastn search of the genes contained in these scaffolds against the barley genome. Of the 4,462 WABMs mapping to the A-genome, hits against the *T. urartu* genomic scaffolds were obtained in 3,918 (87.8%) sequences, and 2,856 (64.0%) were anchored to the barley physical map (Table 3). In total, 1,658 (37.2%) markers were mapped to the same homoeologous group chromosomes (e.g. between 1A and 1H), accounting for ∼40% for most of the chromosomes but only 8.5% for chromosome 4A. Collinearity between the A- and H-genomes was observed for many of these markers (Fig. 4), except on chromosome 4A, where inversions and translocations have been reported.^31,32^ Translocations among chromosomes 4A, 5A, and 7B could also be observed, as previously reported (Supplementary Fig. S3).

**Table 3.** Summary of blastn results against genome sequences

Mapped sequences	Hits against draft genome	Hits against barley genome	Hits on the same homoeologous chromosomes
A genome	3,918 (87.8%)	2,856 (64.0%)	1,658 (37.2%)
1A	464 (84.5%)	379 (69.0%)	265 (48.3%)
2A	488 (91.6%)	338 (63.4%)	238 (44.7%)
3A	458 (87.2%)	333 (63.4%)	238 (45.3%)
4A	851 (85.2%)	608 (60.9%)	85 (8.5%)
5A	416 (91.4%)	315 (69.2%)	184 (40.4%)
6A	555 (85.6%)	409 (63.1%)	293 (45.2%)
7A	686 (91.1%)	474 (62.9%)	355 (47.1%)
B genome	–	3,972 (62.4%)	1,772 (27.8%)
1B	–	629 (63.4%)	279 (28.1%)
2B	–	793 (64.4%)	389 (31.6%)
3B	–	568 (61.3%)	249 (26.9%)
4B	–	290 (58.7%)	150 (30.4%)
5B	–	588 (60.9%)	268 (27.8%)
6B	–	573 (61.9%)	207 (22.4%)
7B	–	531 (63.8%)	230 (27.6%)
D genome	1,319 (59.2%)	1,006 (45.2%)	923 (41.4%)
1D	216 (64.7%)	150 (44.9%)	110 (32.9%)
2D	514 (61.6%)	436 (52.3%)	417 (50.0%)
3D	230 (59.3%)	181 (46.6%)	176 (45.4%)
4D	30 (41.7%)	23 (31.9%)	23 (31.9%)
5D	131 (52.6%)	90 (36.1%)	89 (35.7%)
6D	90 (50.3%)	68 (38.0%)	64 (35.8%)
7D	108 (62.8%)	58 (33.7%)	44 (25.6%)
Total	5,237 (78.3%)	7,834 (60.0%)	4,353 (33.3%)

Sequences mapped on A- and D-genomes were first blast searched against *T. urartu* and *Ae. tauschii* genome sequences and then the obtained scaffolds were assigned to the barley physical map based on their genes. B-genome sequences were directly aligned to the barley genome.

The proportion of D-genome markers with hits against the *Ae. tauschii* genomic sequence (1,319/2,228), and consequently, those anchored on the barley physical map (1,006/2,228), was much lower than the A-genome markers (Table 3). However, a similar percentage (41.4%) was observed for markers mapping to the same homoeologous group chromosomes.

For B-genome markers, the proportion of hits against the barley genome was similar to that in the A-genome (62.4%), but the number of markers mapping to the same homoeologous group was lower in the B-genome (27.8%, Table 3). Although collinearity with the barley genome was observed for many of these markers, the number of non-syntenic markers tended to be higher in the B-genome (Fig. 4). In general, synteny with barley chromosomes at the centromeric region was clearly observed only in a few chromosomes such as 1A and 2A.

### 3.5. QTL analysis for 14 morphological and agricultural traits

To compare the efficiency of the newly constructed map with the original SSR map developed by Kobayashi *et al.*^16^ for analysing complex traits, QTL analysis of 14 agronomically important traits was performed using both maps. Flowering-related traits such as HT, FT, MT, and GFP were evaluated over two growing seasons at Kobe10 and Kobe12 (see Section 2.1.), and at Kyoto12 (Supplementary Table S1). Of the spike-related traits, SpN, SLperSpN (indicative of spike compactness), and T5SpL were evaluated for Kobe12; SL was also evaluated for Kyoto12. Seed-related traits such as SdL, SdW, SdH, and SdLperW were measured for Kobe12. Phenotype data for CL and TN were obtained for Kyoto12. The mean values of the parental lines and RILs are shown in Supplementary Table S8. Strong positive correlations were observed among HT, FT, and MT, and between SL and SLperSpN (Supplementary Table S9). Relatively, strong correlation was also found between T5SpL and SLperSpN, SdH and SdW, and SdLperW and SdW.

QTL analysis was performed using composite interval mapping, and only the QTLs with peaks above the LOD threshold computed by a 1,000-permutation test were selected. The LOD threshold values were significantly higher (*P* < 0.001) in the new high-density map, with an average of 4.76 ± 0.15, than in the original SSR map (3.76 ± 0.08). In total, 29 QTLs were identified using the SSR map and 32 QTLs using the high-density map, of which 25 were identified in both maps (Supplementary Table S10 and Fig. S4); seven QTLs were identified only in the high-density map and four QTLs only in the SSR map.

The 1.5-LOD support interval, meaning the interval in which the LOD score was within 1.5 units of its maximum, tended to be smaller in the high-density map (average of 14.62 ± 18.03 cM) than in the SSR map (22.01 ± 19.85 cM), but the difference was not significant (*P* = 0.129) (Table 4). Excluding the D-genome QTLs, where the map resolution was still low, the difference became significant (9.45 ± 3.29 cM for the high-density map and 17.21 ± 11.29 cM for the SSR map, *P* < 0.01). The number of markers within the 1.5-LOD intervals was also significantly higher (*P* < 0.05) in the high-density map (average of 16.40 ± 22.97) than in the SSR map (average of 4.20 ± 1.98) (Table 4).

**Table 4.** Comparison of 1.5-LOD support intervals and number of markers within this interval between the newly constructed and original SSR maps

QTL^a^	Chr	Newly constructed map		Original SSR map^b^	
		1.5-LOD interval (cM)	Number of markers	1.5-LOD interval (cM)	Number of markers
HT_Kobe10	2B	6.49	4	18.18	3
FT_Kobe10	2B	6.49	4	18.18	3
MT_Kobe10	2B	6.49	4	18.18	3
GFP_Kobe10	1A	9.53	14	9.47	4
HT_Kobe12	2B	9.14	7	18.18	3
HT_Kobe12	5D	73.64	5	76.43	5
FT_Kobe12	2B	9.14	7	18.18	3
FT_Kobe12	5D	73.64	5	76.43	5
MT_Kobe12	2B	6.49	4	18.18	3
HT_Kyoto12	2B	6.49	4	18.18	3
FT_Kyoto12	2B	6.49	4	18.18	3
SL_Kobe12	3A	14.68	10	31.42	4
SL_Kobe12	6A	10.98	23	22.34	7
SL_Kyoto12	4A	10.99	28	12.81	6
SpN_Kobe12	2A	14.68	9	5.80	8
SpN_Kobe12	2B	9.14	7	11.01	2
SLperSpN_Kobe12	4A	9.80	21	12.81	6
SLperSpN_Kobe12	6A	9.67	22	12.00	5
T5SpL_Kobe12	2B	2.31	5	11.01	2
T5SpL_Kobe12	3A	14.68	10	60.77	5
T5SpL_Kobe12	4A	10.99	28	5.34	3
SdH_Kobe12	1B	8.06	45	15.61	10
SdLperW_Kobe12	2D	10.41	115	41.19	3
CL_Kyoto12	2B	15.06	10	11.01	2
TN_Kyoto12	1A	10.03	15	11.74	4
Total average		14.62 ± 18.03	16.40 ± 22.97*	22.91 ± 19.85	4.20 ± 1.97
Average of AB-genome		9.45 ± 3.29**	12.95 ± 10.75***	17.21 ± 11.29	4.18 ± 2.08

^a^Only the 25 QTLs identified in both maps were included.

^b^Kobayashi *et al.*^16^

Student's *t*-test was used to test for statistical significance (*P < 0.05; **P < 0.01; ***P < 0.001).

Most of the identified QTLs were located in a similar chromosomal region to those previously reported (Supplementary Table S10). Of the flowering-related traits, the early flowering effect of the CS *Ppd-B1* allele was observed in all the datasets and explained >30% of the PV. The early flowering effect of CS at 5B and 5D QTL for HT and FT was observed only in one environment (Kyoto12 and Kobe12, respectively), indicating a possible genotype × environment interaction.

Of the spike-related traits, QTLs for SL were identified on chromosomes 3A, 4A, and 6A, and explained ∼10% of the total PV. The CS allele increased SL at the 3A QTL, but decreased it at the 4A and 6A QTLs. The 3A QTL also exhibited an effect on T5SpL (explaining 7.77% of PV) and SLperSpN (9.82% of PV). Similarly, the 4A QTL also affected T5SpL (15.39% of PV) and SLperSpN (10.39% of PV) and might explain the positive correlation between these traits (Supplementary Table S9). The 6A QTL had an effect on SLperSpN explaining 15.01% of the PV. QTLs for SpN were identified on homoeologous group 2 chromosomes, of which 2B QTL (linked to the *Ppd-B1* locus) exhibited a major effect and explained 22.16% of the PV.

A QTL for SdL was detected on chromosome 3A (6.25% of PV), and another was detected for SdH on chromosome 1B (9.60% PV). QTLs for SdW and SdLperW were detected on the long arm of chromosome 2D, and explained, respectively, 5.94 and 8.78% of the total PV. This QTL also might explain the significant correlation between SdW and SdLperW (Supplementary Table S9).

## 4. Discussion

The construction of high-resolution genetic maps in common wheat has required extensive effort due to its large genome and allohexaploid nature. More than 60% of wheat and barley genomes are composed of REs, with retroelements found at least four times more frequently than DNA transposons.^25–27,33^ In this study, a microarray-based polymorphism detection system was developed using NGS data obtained from complexity-reduced genomic DNA samples of CS and M808. Although the reduction in complexity using a combination of two restriction enzymes, *Pst*I and *Bst*NI, did not affect the composition of REs (Supplementary Table S7), most of the array-based markers used for construction of the genetic map were derived from genic regions (Supplementary Table S6). In addition, retroelements were under-represented in the array-based markers, indicating that the selection of useful polymorphic probes decreased the number of sequences derived from highly repetitive regions.

SNP typing of the entire mapping population using NGS technology has also been applied in polyploid wheat species.^11–13^ Although the GBS approach is cost-effective and fast, it also often leads to a significant amount of missing data due to the low coverage of sequencing.^15^ These missing data points have mostly been replaced with estimated genotypes through statistical imputation.^15^ An advantage of the array-based genotyping approach in wheat is the low generation of missing data, in contrast to the GBS approach. In 9 of the 210 RILs (F_7_ generation) of CS and M808, the number of missing data points was higher than the mean + 2 SD, and they were thus excluded. These missing data points can be explained in part by the presence of heterozygous regions, because data of ambiguous intensity were converted into missing values. In the remaining 201 RILs, no missing data were observed for most of the markers (Supplementary Fig. S1B), indicating a high yield of data from this array-based genotyping system.

The high-density genetic map was composed of 13,480 SSR marker and WABMs, and the markers were distributed across the allohexaploid wheat genome, especially in the A- and B-genomes (Fig. 2). The mapped marker number was similar to that constructed using the GBS approach.^11^ As reported in previous studies, the number of markers in the D-genome was much lower than in the A- and B-genomes of common wheat (Table 1).^8,10^ The reads and contigs anchored to the high-density map were also mapped *in silico* against the physical map of barley, because common wheat lacks any physical map. The draft genome sequences of *T. urartu*^26^ and *Ae. tauschii*^25^ were used for the anchoring of A- and D-genome markers, whereas B-genome markers were directly mapped to the barley genome. The use of *T. urartu* and *Ae. tauschii* genome information greatly increased the number of markers aligned on the same homoeologous group chromosomes of barley compared with the B-genome markers (Table 3). Because of the lower number of D-genome markers, collinearity with barley chromosomes was difficult to observe (Fig. 4). In most cases, collinearity was not observed at the centromeric region. This might be due to the lower gene density and higher number of repetitive sequences present in the proximal region of barley and wheat chromosomes.^27,28^

In the high-density map, segregation distortion was observed in six chromosomal regions, with the long arm of chromosome 6B showing the greatest distortion (Fig. 2 and Supplementary Table S4). This might have been caused by the presence of the pollen killer gene, *Ki*, on the long arm of chromosome 6B of CS.^34,35^ *Ki* would affect marker segregation in a large chromosomal region of 6BL, though its position on 6BL has not been accurately determined yet.^35^ Distortions on chromosomes 2B and 5B are due to an epistatic interaction of hybrid necrosis alleles, *Ne1^w^* on the long arm of chromosome 5B of CS, and *Ne2* on the short arm of chromosome 2B of M808.^35^ *Ne1* and *Ne2* have been roughly mapped to 5BL and 2BS, respectively.^36^ The distorted regions of 2BS and 5BL were more limited around the causal genes than on 6BL. The three other segregation distortions on chromosomes 2A, 5A, and 7B were not observed in our previous study using the original SSR map of CS and M808.^35^ Thus, the increased map resolution should clarify the presence of additional regions showing segregation distortion. However, the causal genes for the segregation distortion on chromosomes 2A, 5A, and 7B are unknown. In general, segregation distortion is considered to occur due to the presence of a reproductive barrier gene,^37–39^ suggesting that reproductive barrier genes might be located in these three chromosomal regions.

QTL analysis using the high-density and SSR maps identified a similar number of QTLs for both maps (Supplementary Table S10). Although most of the QTLs were commonly identified in the two maps, a higher number of QTLs was identified in the high-density map. In the high-density map, the LOD threshold values computed by a 1,000-permutation test were significantly higher, as reported in rice.^40^ Thus, the four QTLs identified only in the SSR map might be masked in the high-density map due to these higher threshold values. The increased map resolution resulted in identification of QTLs in a narrower chromosomal region and in a more accurate position than for the SSR map (Table 4 and Supplementary Fig. S4).

In the high-density map, the QTL associated with flowering-related traits corresponded well to the *Ppd-B1* locus on chromosome 2B and the *VRN-D1* locus (Supplementary Fig. S4). The early flowering phenotype of CS (Supplementary Table S8) can be explained mainly by the effect of the photoperiod-insensitive allele of *Ppd-B1* (probably due to its higher copy number)^41,42^ and dominant spring allele of *VRN-D1*.^43^ The spike compactness (SL/SpN) and short SL of CS can be explained by the 6A and 4A QTLs. The 4A QTL also exhibited an effect on T5SpL, which might contribute to the square-head like spike morphology of CS. On the other hand, the CS allele at the 3A QTL contributed to a less compact spike, longer SL, and longer T5SpL. Grain size and shape are associated with milling quality and yield in wheat. Large and spherical grains have been predicted to be the optimum grain morphology to increase milling yield.^44^ The CS allele at the 2D QTL, with an increasing effect on SdW and reduced SdLperW, and the M808 allele at the 1B QTL (increased SdH) contributed in seed roundness. Favourable alleles for increasing yield were also found in CS at the 2B QTL for CL (reduced plant height) and at the 1A QTL for TN (increased number of tillers with ears). Therefore, the results of QTL analyses indicate that the high-density map efficiently and precisely functions to identify QTLs for morphological and agricultural traits.

Because of the difference in geographical origin and growth habit of the common wheat cultivars CS (spring habit Chinese variety) and M808 (winter habit Ukrainian variety) used for the development of our array-based system, it is expected to work better between spring and winter wheat and/or Chinese/Asian and European variety. Further study is needed to test the usefulness of this genotyping system using a large wheat population set.

A number of tightly linked WABMs were found in most of the QTLs, which could facilitate marker-assisted selection or map-based cloning of genes. For this application, the marker of interest should be converted into a PCR marker. Our results demonstrated that this array-based polymorphism detection system can yield high-quality genotype data useful for high-density genetic map construction and QTL analysis of agronomically important traits.

## Supplementary Data

Supplementary data are available at www.dnaresearch.oxfordjournals.org.

## Funding

This work was supported by a grant from the Ministry of Education, Culture, Sports, Science and Technology (MEXT) of Japan [Grant-in-Aid for Scientific Research (B) no. 25292008] to S.T., by cooperative research funds from TOYOTA Motor Co. Ltd., and by Special Coordination Funds for Promoting Science and Technology, Creation of Innovation Centers for Advanced Interdisciplinary Research Areas (Innovative BioProduction Kobe), MEXT, Japan. Funding to pay the Open Access publication charges for this article was provided by a grant from the Ministry of Education, Culture, Sports, Science and Technology of Japan [Grant-in-Aid for Scientific Research (B) No. 25292008] to S.T.

## Supplementary Material

Supplementary Data
