# Supplementary material for: A High-Density Genetic Map with Array-Based Markers Facilitates Structural and Quantitative Trait Locus Analyses of the Common Wheat Genome
Source: DNA Res. 2014 Jun 27;21(5):555–67. doi: 10.1093/dnares/dsu020 (PMC4195500; doi:10.1093/dnares/dsu020)
Supplement: Supplementary Data [file supp_21_5_555__index.html]

A High-Density Genetic Map with Array-Based Markers Facilitates Structural and Quantitative Trait Locus Analyses of the Common Wheat Genome — A High-Density Genetic Map with Array-Based Markers Facilitates Structural and Quantitative Trait Locus Analyses of the Common Wheat Genome — Supplementary Data 

# A High-Density Genetic Map with Array-Based Markers Facilitates Structural and Quantitative Trait Locus Analyses of the Common Wheat Genome

## Supplementary Data

Supplementary Data

**Files in this Data Supplement:**

- Supplementary suppFigures - pdf file
- Supplementary TableS1S2S6S7S8 - pdf file
- Supplementary TableS9 - pdf file
- Supplementary TableS3S4S5S10 - xls file
