# Supplementary material for: A High-Density Genetic Map with Array-Based Markers Facilitates Structural and Quantitative Trait Locus Analyses of the Common Wheat Genome
Source: DNA Res. 2014 Jun 27;21(5):555–67. doi: 10.1093/dnares/dsu020 (PMC4195500; doi:10.1093/dnares/dsu020)
Supplement: Supplementary Data [file supp_dsu020_dsu020supp_suppFigs.pdf]

(a)

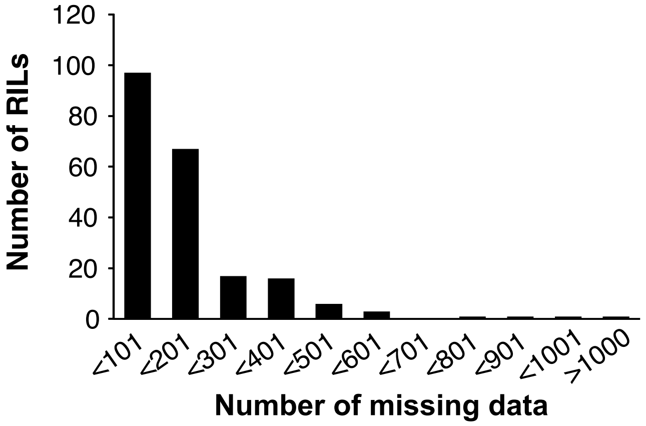

(b)

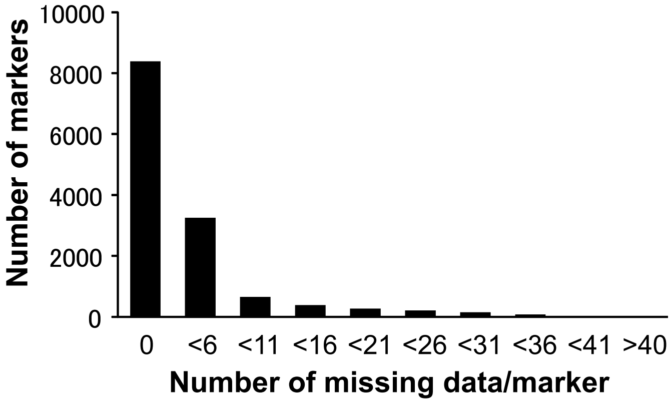

Supplementary Figure S1. Distribution of missing data. Histograms represent (a) the number of missing data points per RIL for 15,530 markers and (b) the number of missing data points per marker for 201 RILs.

(a)

(b)

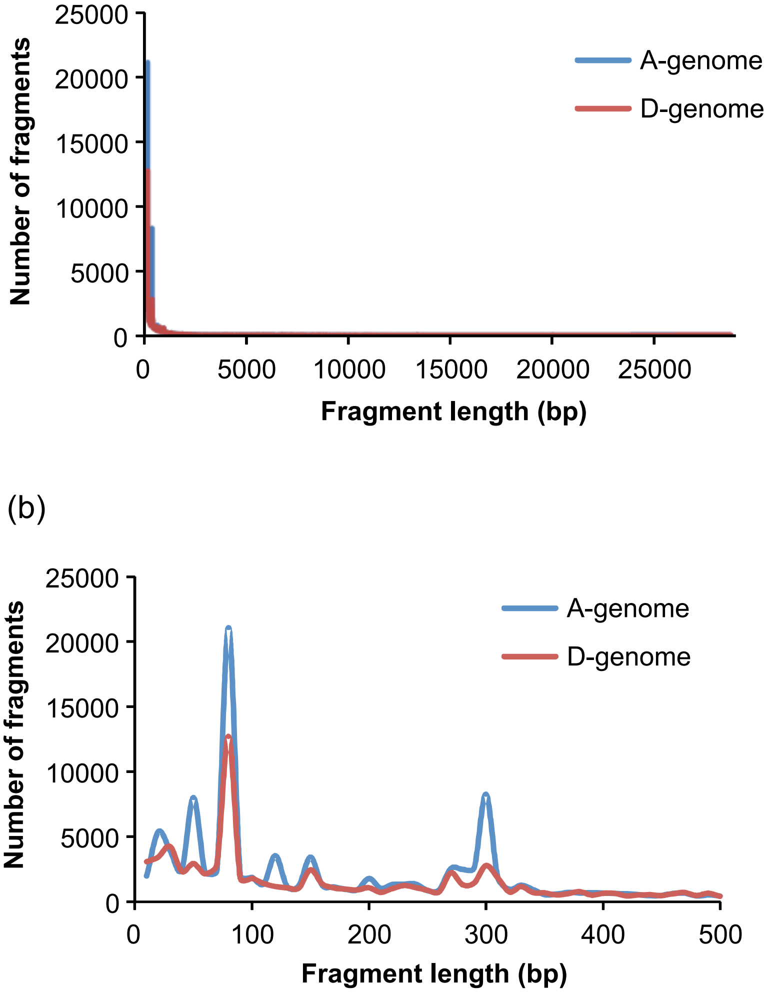

Supplementary Figure S2. Size distribution of *in silico* digested A- and D-genome fragments. Genomic scaffolds of *T. urartu* and *Ae. tauschii* were *in silico* digested with *Pst*I and *Bst*NI and fragments having only *Pst*I sites at the both ends were extracted. Size distribution of (a) all the extracted DNA fragments and (b) fragments ≤500 bp.

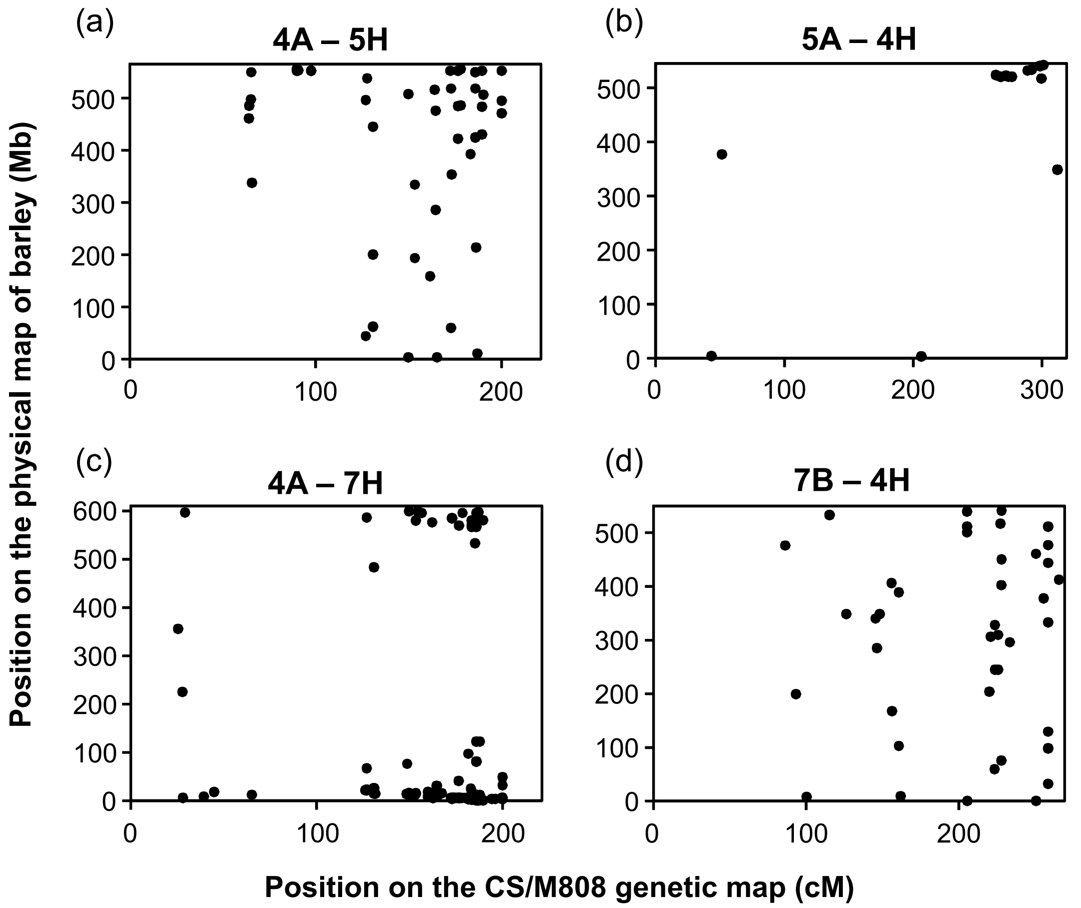

Supplementary Figure S3. Evidence of translocations involving chromosomes 4A, 5A and 7B of common wheat. Markers on chromosome 4AL were also mapped to chromosomes 5HL (a) and on 5AL to chromosome 4HL (b), which might correspond to the region of 4AL-5AL reciprocal translocation. In contrast, the probable 4AL-7BS translocation was observed in a 4A-7H comparison (c) but not in a 7B-4H comparison (d). The *y*-axis indicates the position on the physical map of barley in Mb and the *x*-axis the position on the genetic map of wheat in cM.

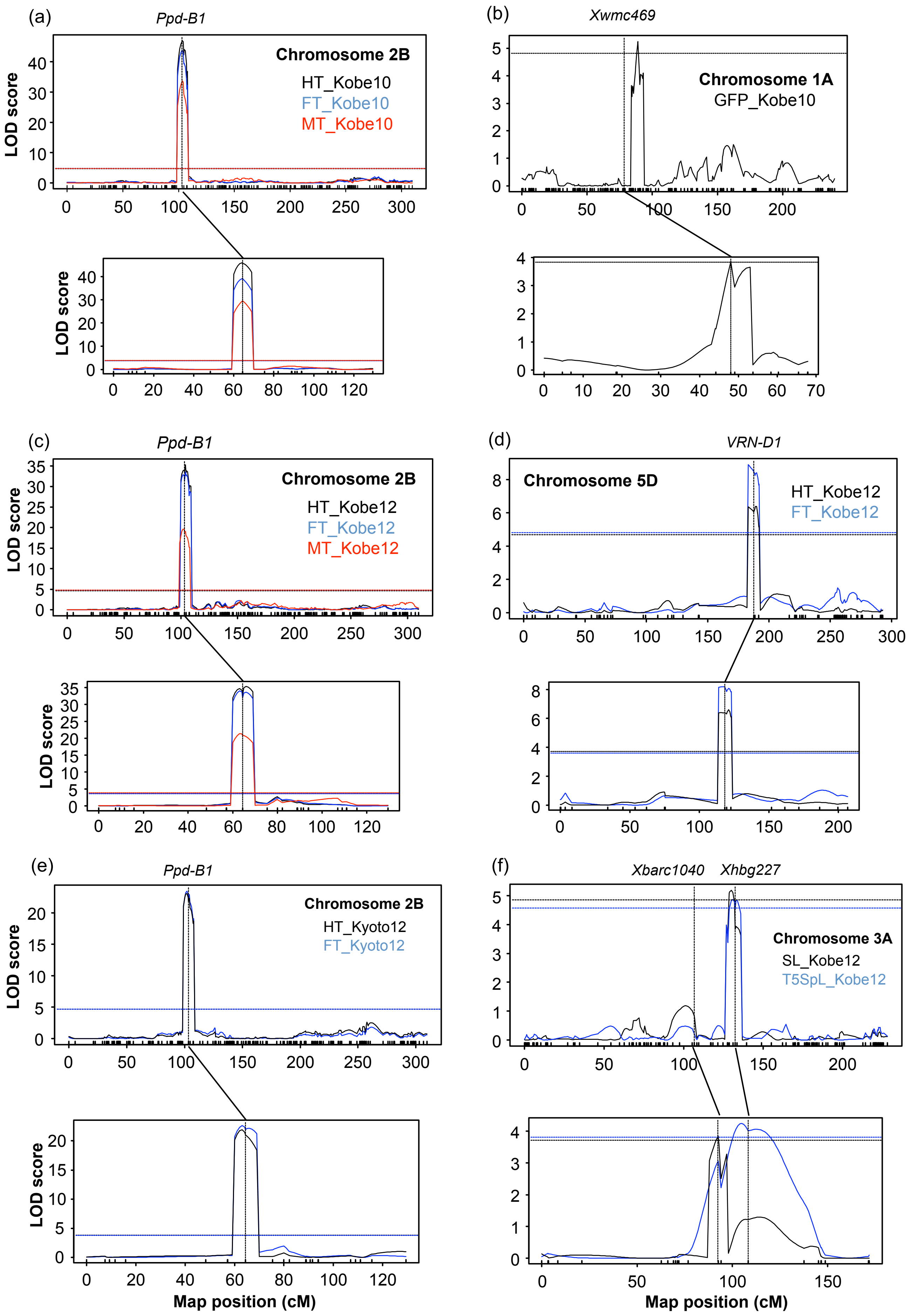

Supplementary Figure S4. Comparison of LOD curves of the QTLs identified in the high-density map and SSR map. Only the 25 QTLs commonly identified in both maps are shown: QTLs for flowering-related traits (a–e), spike-related traits (f–j), seed-related traits (k–l), CL (m) and TN (n). The locations of the QTLs were compared using SSR or gene specific markers, indicated by vertical lines. Horizontal lines indicate the LOD threshold values computed by a 1,000-permutation test.

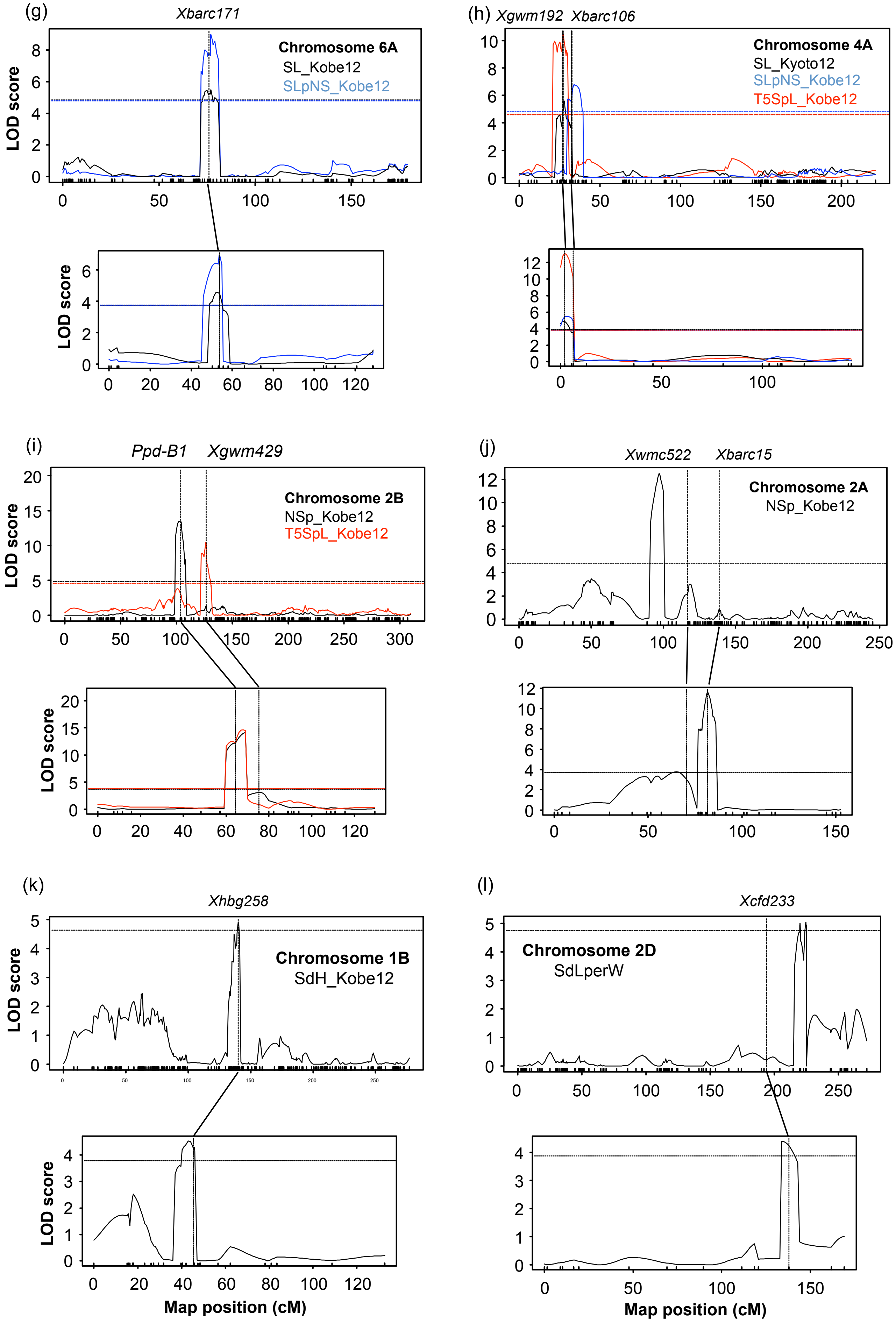

Supplementary Figure S4 (cont.)

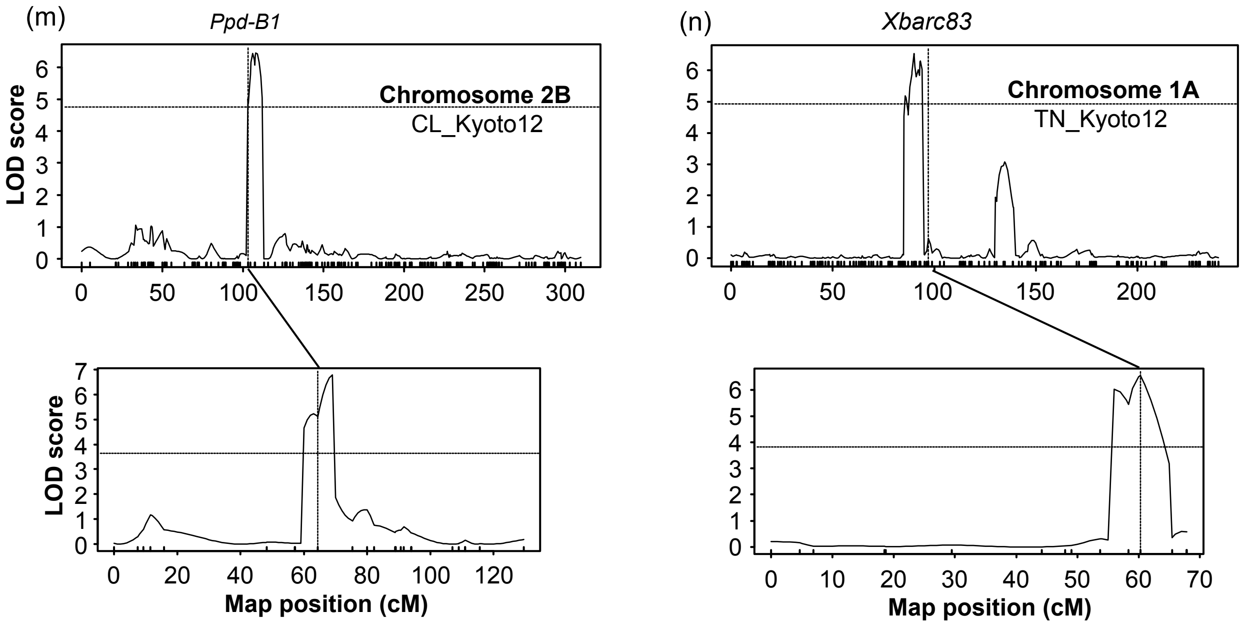

Supplementary Figure S4 (cont.)
