# Supplementary material for: A High-Density Genetic Map with Array-Based Markers Facilitates Structural and Quantitative Trait Locus Analyses of the Common Wheat Genome
Source: DNA Res. 2014 Jun 27;21(5):555–67. doi: 10.1093/dnares/dsu020 (PMC4195500; doi:10.1093/dnares/dsu020)
Supplement: Supplementary Data [file supp_dsu020_dsu020supp_TableS1S2S6S7S8.pdf]

Supplementary Table S1. List of evaluated traits

Traits	Location (growing season)
Flowering-related traits	
HT, FT	Kobe (2009-2010; 2011-2012), Kyoto (2011-2012)
MT, GFP	Kobe (2009-2010; 2011-2012)
Spike related-traits	
SL	Kobe (2011-2012), Kyoto (2011-2012)
SpN, SLperSpN	Kobe (2011-2012)
T5SpL	Kobe (2011-2012)
Seed-related traits	
SdL, SdW, SdH, SdLperW	Kobe (2011-2012)
Other traits	
CL	Kyoto (2011-2012)
TN	Kyoto (2011-2012)

Supplementary Table S2. Total sequence/read length and genome coverage at each step of markers development

Step of markers development	CS's length*	M808's length*	Total length*
Raw reads	7.77 Gb (0.46x)	7.59 Gb (0.45x)	15.36 (0.90x)
Filtered reads	6.04 Gb (0.36x)	6.09 Gb (0.36x)	12.13 (0.71x)
Contigs + unused reads	5.01 Gb (0.29x)	4.69 Gb (0.28x)	9.70 Gb (0.57x)
Primary array	38.87 Mb (2.29×10^{-3}x)	37.70 Mb (2.22×10^{-3}x)	76.57 Mb (4.51×10^{-3}x)
Secondary array	5.84 Mb (3.43×10^{-4}x)	6.83 Mb (4.02×10^{-4}x)	12.67 Mb (7.45×10^{-4}x)
Mapped sequences	2.64 Mb (1.55×10^{-4}x)	2.92 Mb (1.72×10^{-4}x)	5.56 Mb (3.27×10^{-4}x)

*Genome coverage, given in parenthesis, was calculated considering the wheat genome size of 17 Gb.

Supplementary Table S6. Number of probes with hits against genes and REs in each genome

Genome	Number of total markers	Hits against genic sequences	Hits against REs
A-genome	4,462	1765 (39.6%)	492 (11.0%)
B-genome	6,366	2517 (39.5%)	736 (11.6%)
D-genome	2,228	908 (40.8%)	256 (11.5%)
Total	13,056	5,190 (39.8%)	1484 (11.4%)

Supplementary Table S7. Number of *in silico* digested A- and D-genome fragments with hits against REs

REs	A-genome	D-genome
DNA transposons	14,353 (17.0%)	16,196 (26.8%)
CACTA	12,424 (86.6%)	14,182 (87.6%)
MITE	665 (4.6%)	657 (4.1%)
Tc1/Mariner	358 (2.5%)	321 (2.0%)
Other DNA transposons	289 (2.0%)	305 (1.9%)
Mutator	394 (2.7%)	500 (3.1%)
Harbinger	233 (1.6%)	231 (1.4%)
Retroelements	68,968 (81.7%)	42,642 (70.5%)
LTR/Gypsy	36,416 (52.8%)	18,579 (43.6%)
Other LTRs	26,661 (38.7%)	18,715 (43.9%)
LTR/Copia	4,982 (7.2%)	4,247 (10.0%)
LINE	885 (1.3%)	1,084 (2.5%)
SINE + unclassified non-LTR retrotransposons	24 (0.03%)	17 (0.04%)
Other REs	1,088 (1.3%)	1,683 (2.8%)
**Total**	**84,410**	**60,522**

Supplementary Table S8. Parental and RIL population means for evaluated traits

	CS	M808	RIL population
HT_Kobe10	-	-	151.17 ± 7.50 days
FT_Kobe10	-	-	158.22 ± 5.63 days
MT_Kobe10	-	-	191.37 ± 5.45 days
GFP_Kobe10	-	-	33.15 ± 3.35 days
HT_Kobe12	151.33 ± 0.58*** days	163.33 ± 0.58 days	159.54 ± 4.13 days
FT_Kobe12	157.33 ± 0.58*** days	167.67 ± 0.58 days	164.20 ± 3.42 days
MT_Kobe12	189.33 ± 0.58*** days	198.33 ± 1.53 days	193.99 ± 4.06 days
GFP_Kobe12	32.00 ± 0.00 days	30.67 ± 1.15 days	29.83 ± 2.44 days
HT_Kyoto12	-	-	183.14 ± 4.12 days
FT_Kyoto12	-	-	184.91 ± 3.70 days
SL_Kobe12	93.34 ± 1.90*** mm	123.41 ± 3.44 days	102.92 ± 12.67 days
SL_Kyoto12	-	-	119.18 ± 16.04 days
SpN	24.67 ± 0.58	25.00 ± 0.00	23.90 ± 1.77
SLperSpN	3.79 ± 0.16*** mm	4.94 ± 0.14 mm	4.31 ± 0.49 mm
T5SpL	16.58 ± 0.61*** mm	25.05 ± 1.48 mm	21.50 ± 4.70 mm
SdL	6.34 ± 0.23*** mm	7.02 ± 0.06 mm	6.77 ± 0.36 mm
SdW	3.23 ± 0.09*** mm	3.35 ± 0.06 mm	3.36 ± 0.23 mm
SdH	3.00 ± 0.09*** mm	3.35 ± 0.08 mm	3.01 ± 0.17 mm
SdLperW	1.96 ± 0.07***	2.10 ± 0.05	2.02 ± 0.16
CL	-	-	113.50 ± 12.61 cm
TN	-	-	12.66 ± 2.81

Data are means ± SD.

Student's *t*-test was used to test for statistical significance ($^{*}P < 0.05$; $^{**}P < 0.01$; $^{***}P < 0.001$).
