# Supplementary material for: A High-Density Genetic Map with Array-Based Markers Facilitates Structural and Quantitative Trait Locus Analyses of the Common Wheat Genome
Source: DNA Res. 2014 Jun 27;21(5):555–67. doi: 10.1093/dnares/dsu020 (PMC4195500; doi:10.1093/dnares/dsu020)
Supplement: Supplementary Data [file supp_dsu020_dsu020supp_TableS9.pdf]

Supplementary Table S9. Pearson's correlation coefficient (*r*) among the evaluated traits

	FT*	MT*	GFP*	SL_Kobe	SL_Kyoto	SpN	SL/SpN	T5SpL	SdL	SdW	SdH	SdL/W	CL	TN
HT*	0.980c	0.805c	-0.337c	0.000	0.124	0.446c	-0.290c	-0.401c	0.054	-0.147a	-0.118	0.182b	0.275c	-0.052
FT*		0.817c	-0.351c	0.021	0.154a	0.479c	-0.288c	-0.406c	0.055	-0.146a	-0.109	0.178a	0.278c	-0.021
MT*			0.253c	0.067	0.179a	0.368c	-0.164a	-0.328c	0.108	0.026	0.010	0.042	0.250c	-0.080
GFP*				0.073	0.033	-0.207b	0.216b	0.148a	0.083	0.287c	0.200b	-0.232c	-0.058	-0.096
SL_Kobe					0.823c	0.415c	0.809c	0.465c	0.480c	0.273c	0.064	0.053	0.059	-0.066
SL_Kyoto						0.410c	0.623c	0.413c	0.429c	0.187b	0.061	0.104	0.109	-0.026
SpN							-0.195b	-0.323c	0.129	0.101	-0.011	-0.029	0.285c	-0.051
SL/SpN								0.711c	0.445c	0.245c	0.091	0.067	-0.116	-0.044
T5SpL									0.260c	0.069	0.052	0.119	-0.200b	0.024
SdL										0.289c	0.293c	0.387c	0.136	-0.128
SdW											0.636c	-0.764c	0.146a	-0.154a
SdH												-0.412c	0.018	-0.080
SdL/W													-0.058	0.051
CL														-0.248c

*HT, FT, MT and GFP are represented by the data obtained at Kobe10.

Significances of relationships are indicated by letters: a ($P < 0.05$), b ($P < 0.01$) and c ($P < 0.001$).
